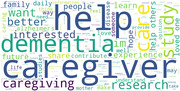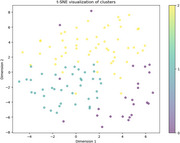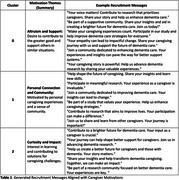# Advancing Dementia Research Recruitment with AI‐Driven Insights and Behavioral Nudges

**DOI:** 10.1002/alz70858_103883

**Published:** 2025-12-26

**Authors:** Jessica Andrea Hernandez Chilatra, Wesley R Browning, Xiaoqian Jiang, Carolyn Pickering

**Affiliations:** ^1^ University of Texas Health Science Center at Houston, Houston, TX, USA

## Abstract

**Background:**

Recruitment of family caregivers and persons with dementias into research often presents unique challenges that require innovative strategies. Behavioral “nudges,” based on choice architecture from behavioral economics, offer a non‐coercive way to structure choices and encourage participation. This study leverages AI‐driven methods, large language models (LLM), advanced natural language processing (NLP) pipelines, and behavioral nudge theory as a framework to analyze motivations and develop recruitment messaging strategies tailored to caregivers.

**Method:**

Open‐ended responses from 117 caregivers, primarily middle‐aged women reflecting diverse caregiving experiences, were analyzed using NLP techniques. Textual data was embedded via pre‐trained BERT models to capture semantic meaning. Subsequently, K‐means clustering was employed to identify patterns within the data, revealing distinct thematic motivations among caregivers. Behavioral nudge theory was the conceptual framework for analyzing motivations and designing recruitment messages. GPT‐4o, an advanced LLM, was employed to process theoretical literature, extract principles, and generate culturally sensitive, non‐coercive messaging aligned with caregivers' motivations.

**Result:**

Thematic analysis revealed three primary motivation clusters: (1) a desire to contribute to research for altruistic or scientific advancement purposes, (2) personal experiences with caregiving and a need for emotional support, and (3) curiosity about dementia care and management strategies. A total of 60 messages were generated incorporating social proof, empathetic framing, and loss aversion techniques with high contextual relevance and potential engagement. For example, messages framing the role of caregivers in shaping future dementia care had high engagement potential. Behavioral insights also highlighted that culturally sensitive and empathetic messaging is required for diverse caregiver populations.

**Conclusion:**

This study demonstrates how integrating behavioral nudge theory, LLM, and advanced NLP techniques improves the recruitment of caregivers into dementia research studies. We provide a scalable, ethical, and practical approach to addressing longstanding recruitment challenges by aligning recruitment messages with identified motivations. These findings underscore the value of leveraging AI‐driven insights and behavioral frameworks in designing non‐coercive interventions to support caregiver research participation.